# Early Decline in Serum Haptoglobin During Impella CP Support Compared With Intra‐Aortic Balloon Pump in Cardiogenic Shock: A Single‐Center Observational Study

**DOI:** 10.1161/JAHA.126.049038

**Published:** 2026-08-07

**Authors:** Shoji Kawakami, Daisuke Kawakami, Masato Takei, Yoshihiro Ota, Yosuke Matsumoto, Shihomi Nawata, Eigo Nawata, Hidenobu Koga, Jun‐Ichiro Nishi

**Affiliations:** ^1^ Department of Cardiology Aso Iizuka Hospital Fukuoka Japan; ^2^ Division of Cardiac Care Unit, Advanced Emergency Medical Services Center Kurume University Hospital Fukuoka Japan; ^3^ Division of Cardiovascular Medicine, Department of Internal Medicine Kurume University School of Medicine Fukuoka Japan; ^4^ Department of Intensive Care Medicine Aso Iizuka Hospital Fukuoka Japan; ^5^ Clinical Research Support Office Aso Iizuka Hospital Fukuoka Japan

**Keywords:** haptoglobin, hemolysis, Impella CP, intra‐aortic balloon pumping, venoarterial extracorporeal membrane oxygenation, Cardiopulmonary Resuscitation and Emergency Cardiac Care

## Abstract

**Background:**

Hemolysis is a recognized complication of mechanical circulatory support and may contribute to acute kidney injury in patients with cardiogenic shock. Early hemolysis during Impella support, particularly compared with intra‐aortic balloon pump (IABP), remains incompletely characterized.

**Methods:**

We retrospectively analyzed a single‐center registry of consecutive patients with cardiogenic shock treated with IABP or Impella between August 2022 and October 2024. Serum haptoglobin was measured at intensive care unit admission and every 12 hours during device support. Low haptoglobin was defined as <19 mg/dL.

**Results:**

Among 91 patients (IABP, n=67; Impella CP, n=24), baseline haptoglobin levels were similar. Patients supported with Impella exhibited a significantly greater decline in haptoglobin beginning 12 hours after intensive care unit admission, particularly in the absence of concomitant venoarterial extracorporeal membrane oxygenation. Low haptoglobin occurred more frequently with Impella than with IABP (67% versus 31%, *P*=0.004), whereas the incidence of overt clinical hemolysis was low and comparable between devices. Impella use (odds ratio, 2.82 [95% CI, 1.45–5.95]; *P*=0.001), concomitant venoarterial extracorporeal membrane oxygenation support (odds ratio, 16.52 [95% CI, 4.65–174.56]; *P*<0.001), and lower mean arterial pressure at intensive care unit admission (OR, 0.95 [95% CI, 0.89–0.99]; *P*=0.006) were independently associated with low haptoglobin.

**Conclusions:**

In patients with cardiogenic shock, Impella CP support was associated with earlier and more frequent decline in serum haptoglobin compared with IABP, suggesting more frequent subclinical hemolysis. Although overt hemolysis was uncommon, early monitoring using serial serum haptoglobin measurements may help inform device management strategies.

**Nonstandard Abbreviations and Acronyms**
AKI: acute kidney injury
ACT: activated clotting time
CS: cardiogenic shock
DanGer Shock: Danish–German Cardiogenic Shock
IABP: intra‐aortic balloon pump
MCS: mechanical circulatory support
SCAI: Society for Cardiovascular Angiography and Interventions
V‐A ECMO: venoarterial extracorporeal membrane oxygenation

**Clinical Perspective**

**What Is New?**

- In patients with cardiogenic shock, Impella CP support was associated with earlier and more frequent subclinical hemolysis than intra‐aortic balloon pump support, with haptoglobin decline becoming evident within 12 hours after intensive care unit admission despite similar baseline levels.
- Concomitant venoarterial extracorporeal membrane oxygenation was strongly associated with low haptoglobin, with similarly low haptoglobin levels observed regardless of the unloading device used.

**What Are the Clinical Implications?**

- Early serial monitoring of serum haptoglobin may help detect subclinical hemolysis before overt clinical hemolysis develops and may help guide timely optimization of device management.

The prognosis of patients with cardiogenic shock (CS) remains poor.^1^, ^2^, ^3^, ^4^ Randomized controlled trials have failed to demonstrate a survival benefit of intra‐aortic balloon pump (IABP) or venoarterial extracorporeal membrane oxygenation (V‐A ECMO) in this population.^2^, ^3^ More recently, the DanGer Shock (Danish–German Cardiogenic Shock) trial demonstrated that circulatory support with Impella CP (Abiomed, Danvers, MA) improves survival in selected patients with CS. However, Impella support is associated with device‐related complications, including bleeding, hemolysis, and limb ischemia.^4^, ^5^ These adverse events may offset the hemodynamic benefits of potent ventricular unloading, and several studies have reported that Impella use is not consistently associated with improved outcomes.^6^, ^7^, ^8^ Among these complications, acute kidney injury (AKI) occurs at a relatively high frequency in patients supported with Impella, and device‐induced hemolysis has been suggested as a potential contributing mechanism.^9^ IABP is generally considered to be associated with less hemolysis than microaxial flow pumps^5^; however, hemolysis has been reported as a recognized, though infrequent, complication of IABP support. Nevertheless, few studies have directly and systematically compared hemolysis between Impella and other mechanical circulatory support (MCS) devices.

Serum haptoglobin has long been used as a diagnostic marker of hemolytic anemia^10^, ^11^ and has also been reported to be useful for detecting mechanical hemolysis during MCS support.^12^, ^13^ In the present study, we longitudinally evaluated hemolysis using serial serum haptoglobin measurements in patients supported with IABP or Impella and explored clinical factors associated with the development of hemolysis.

## Methods

The data that support the findings of this study are available from the corresponding author upon reasonable request, subject to institutional and ethical restrictions. The analytic methods are described in this article and its Supplemental Material. No additional study materials are available. This study was approved by the Ethics Committee of Aso Iizuka Hospital (No. 22066) and was conducted in accordance with the Declaration of Helsinki. The requirement for informed consent was waived due to the study design.

### Study Design

We retrospectively analyzed data from a single‐center registry of consecutive patients admitted to the intensive care unit (ICU) of Aso Iizuka Hospital between August 2022 and October 2024. This registry includes patients who required advanced critical care management, including MCS, mechanical ventilation, renal replacement therapy, targeted temperature management, or other intensive care interventions, as judged by the treating physicians. For the present analysis, we identified patients treated with IABP or Impella, with or without concomitant V‐A ECMO, and classified them according to the first IABP or Impella used during the index ICU admission. Patients who received isolated V‐A ECMO support, had postcardiotomy CS, were on chronic maintenance dialysis, or died or had a do‐not‐resuscitate order within 24 hours of ICU admission were excluded. Shock severity was categorized according to the Society for Cardiovascular Angiography and Interventions (SCAI) classification.^14^ Patients who received prophylactic MCS for high‐risk percutaneous coronary intervention or because of a high perceived risk of hemodynamic deterioration were classified as SCAI stage A.

Aso Iizuka Hospital is a tertiary teaching hospital with 24‐hour catheterization services managed by cardiologists. The ICU is a mixed medical–surgical unit staffed by intensivists. Patients presenting to the emergency department with suspected cardiovascular disease were jointly evaluated by emergency department physicians and cardiologists. Cardiologists determine the need for emergency catheterization, perform percutaneous coronary intervention, and insert MCS. After ICU admission, patient care was comanaged by intensivists and cardiologists. This study was conducted as a retrospective observational analysis using data obtained from routine clinical management. Clinical data were collected from electronic medical records. Shock severity was assessed using the SCAI.^14^

### Management of MCS Devices

All MCS devices were inserted in the catheterization laboratory. IABP support was initiated at a 1:1 counterpulsation ratio. During IABP support, all patients received systemic anticoagulation with unfractionated heparin, and anticoagulation intensity was monitored using activated partial thromboplastin time or activated clotting time (ACT).

During Impella support, the performance level was adjusted to achieve adequate circulatory support according to each patient's hemodynamic status while avoiding suction alarms. In cases of insufficient support flow, device positioning, volume status, and afterload were immediately assessed. All patients received a purge solution containing unfractionated heparin; bicarbonate‐based purge solutions were not used. Systemic anticoagulation was supplemented when necessary, and anticoagulation intensity was monitored using ACT.

During V‐A ECMO support, a 21‐Fr inlet cannula and a 16.5‐Fr outlet cannula were used as the standard initial configuration, with adjustments made according to patient body size and hemodynamic condition. ECMO flow was titrated to achieve adequate circulatory support while avoiding excessive left ventricular afterload. Prepump venous drainage line (suction) pressure was maintained above −100 mm Hg, and total pump head was kept below 400 mm Hg to avoid excessive circuit resistance. When these parameters deviated from the target range or when chattering of the inlet cannula was observed, cannula position, volume status, and afterload were immediately assessed. All patients received systemic anticoagulation, and anticoagulation intensity was monitored using ACT.

Detailed anticoagulation management was based on ACT or activated partial thromboplastin time depending on the device used and clinical condition. ACT was measured every 6 hours and maintained within a target range of 160 to 180 seconds. When activated partial thromboplastin time was used, the target range was set at 1.5 to 2.5 times the baseline value. Anticoagulation intensity was temporarily reduced or withheld in the presence of clinically significant bleeding, according to the clinician's judgment.

In our institution, the management of suspected hemolysis was guided by a standardized institutional protocol. When hemolysis was suspected, device‐related and hemodynamic factors were systematically assessed, including optimization of the pump performance level to avoid suction events, reassessment of device position, evaluation of volume status, and adjustment of afterload. Right ventricular function was also evaluated when appropriate. Intravenous haptoglobin administration was not performed in this cohort. Serum haptoglobin levels were available within ≈1 hour after sample submission and were used as a reference in clinical decision making; thus, management was not performed in a blinded manner. Patient care was provided by a multidisciplinary team consisting of at least 1 cardiologist and 1 intensivist at all times, which contributed to consistent management across cases. Device weaning was performed according to predefined hemodynamic and clinical criteria.

### Assessment of Hemolysis

Serum haptoglobin was measured using an immunoturbidimetric assay without phenotype typing (Nittobo Medical Co., Ltd., Tokyo, Japan). At our institution, the reference range for haptoglobin is 19 to 170 mg/dL, with values <19 mg/dL considered abnormally low. Measurements were obtained at ICU admission (defined as time 0) and every 12 hours during any form of device support, for up to 7 days, corresponding to the recommended duration of Impella CP use in Japan. Total bilirubin, alanine aminotransferase, lactate dehydrogenase, and serum creatinine levels were measured every 24 hours, with results typically available within ≈1 hour. Plasma‐free hemoglobin was not available for clinical use at our institution. Low haptoglobin was defined as a minimum serum haptoglobin level <19 mg/dL, and clinical hemolysis was defined as low haptoglobin in combination with a maximum total bilirubin >2.0 mg/dL during device support. AKI was defined according to the Kidney Disease: Improving Global Outcomes criteria.

### Statistical Analysis

Statistical analyses were performed using JMP version 18 (SAS Institute Inc., Cary, NC). Baseline characteristics, clinical outcomes, and hemolysis‐related biomarkers were compared between patients treated with IABP and those treated with Impella. Categorical variables were reported as frequencies with proportions and were compared using the χ^2^ test or Fisher's exact test, depending on data distribution. Continuous variables were expressed as means±SDs or medians with interquartile ranges and were compared using 1‐way analysis of variance or the Kruskal–Wallis test.

In addition, time‐to‐event analyses were performed to evaluate the temporal patterns of hemolysis‐related outcomes. The cumulative incidence of low haptoglobin and clinical hemolysis during device support was estimated using the Kaplan–Meier method and compared between groups using the log‐rank test. For low haptoglobin, the event was defined as the first occurrence of serum haptoglobin <19 mg/dL. For clinical hemolysis, the event was defined as the time point at which both low haptoglobin and total bilirubin >2.0 mg/dL had been met; when these criteria were met at different time points, the later time point was used. Patients without events were censored at the last available measurement during device support, which ended at device removal, death, or 7 days after ICU admission, whichever occurred first.

To examine whether each variable predicted low haptoglobin during device support, Firth's penalized logistic regression model was applied. A hypothesis‐driven model was constructed using age, male sex, SCAI shock stage D or E before MCS introduction, Impella use, concomitant V‐A ECMO use, mean arterial pressure at ICU admission, and maximum level of alanine aminotransferase during device support. These variables were selected on the basis of a directed acyclic graph informed by clinical reasoning and prior evidence (Figure S1–S4). As a sensitivity analysis, we restricted the analysis to patients with SCAI shock stages B through E to evaluate the robustness of the findings in a more clinically homogeneous population. Missing data were handled by using all available cases for each analysis, and no imputation was performed.

## Results

During the study period, 103 patients with CS received MCS and were admitted to the ICU at our institution. Of these, 3 patients received isolated V‐A ECMO support, 6 were on chronic maintenance dialysis, and 3 died or had a do‐not‐resuscitate order within 24 hours of ICU admission. After these exclusions, 91 patients were included in the present analysis (IABP group, n=67; Impella group, n=24). All patients in the Impella group were treated with Impella CP. The median age was 74 (interquartile range, 69–79) years, and 68 patients (75%) were men. The most common pathogenesis of CS was acute myocardial infarction, observed in 76 patients (84%). Table 1 summarizes the clinical characteristics of patients who received IABP or Impella. Compared with patients who received IABP, those who received Impella had a significantly lower prevalence of dyslipidemia and were more likely to present with advanced CS. Patients who received Impella were also more frequently supported with mechanical ventilation, and their heart rates at ICU admission were significantly higher. Table 2 summarizes ICU management and clinical outcomes of patients who received IABP or Impella. Compared with patients who received IABP, those who received Impella had a significantly higher incidence of AKI. However, neither the occurrence of more severe AKI nor the need for renal replacement therapy differed significantly between the 2 groups. The ICU length of stay was significantly longer among patients who received Impella, whereas ICU survival and 30‐day survival rates were similar between the 2 groups.

**Table 1:** Comparison of the Clinical Characteristics of Patients Who Received IABP or Impella

	Overall	IABP	Impella	Missing	*P* value
Number	91	67	24		
Patient characteristics					
Age, y	74 (69–79)	74 (66–79)	76 (71–79)	0	0.543
Male sex	68 (75)	49 (73)	19 (79)	0	0.785
Body mass index	22.6 (21.0–24.8)	22.6 (21.4–25.7)	21.7 (20.6–23.8)	0	0.187
Body surface area, m^2^	1.64 (1.54–1.74)	1.64 (1.51–1.74)	1.65 (1.54–1.75)	0	1.000
Hypertension	73 (80)	54 (81)	19 (79)	0	1.000
Dyslipidemia	59 (65)	49 (73)	10 (42)	0	0.012
Diabetes	49 (54)	35 (52)	14 (58)	0	0.607
Smoking habit	55 (60)	43 (64)	12 (50)	0	0.223
Chronic kidney disease	25 (27)	15 (22)	10 (42)	0	0.069
Classification of cardiogenic shock				0	0.250
Acute myocardial infarction	76 (84)	53 (79)	23 (96)		
De novo heart failure	6 (7)	5 (7)	1 (4)		
Acute‐on‐chronic heart failure	4 (4)	4 (6)	0 (0)		
Secondary	5 (5)	5 (7)	0 (0)		
Patient status before MCS introduction					
SCAI shock stage				0	0.026
A	23 (25)	22 (33)	1 (4)		
B	15 (16)	11 (16)	4 (17)		
C	15 (16)	11 (16)	4 (17)		
D	18 (20)	9 (13)	9 (38)		
E	20 (22)	14 (21)	6 (25)		
pH	7.35 (7.28–7.42)	7.36 (7.29–7.43)	7.33 (7.27–7.41)	9	0.374
Lactate, mg/dL	25.2 (16.9–55.0)	23.7 (15.2–49.4)	28.0 (19.4–68.6)	9	0.108
Glasgow Coma Scale <8	24 (26)	21 (31)	3 (13)	0	0.105
Concomitant venoarterial extracorporeal membrane support	25 (27)	18 (27)	7 (29)	0	0.797
Patient status at ICU admission					
Mechanical ventilation	63 (69)	42 (63)	21 (88)	0	0.037
Mean arterial pressure, mm Hg	84 (72–97)	86 (72–101)	78 (71–89)	0	0.122
Heart rate, beats per minute	86 (73–102)	80 (71–98)	99 (85–108)	0	<0.001
Mean pulmonary artery pressure, mm Hg (n=71)	19 (13–24)	18 (12–24)	20 (16–25)	0	0.252
Mean right arterial pressure, mm Hg (n=71)	7 (4–10)	8 (4–10)	7 (4–11)	1	0.869
Pulmonary artery pulsatility index (n=71)	2.6 (1.3–5.6)	2.4 (1.2–5.8)	3.0 (2.0–5.9)	1	0.327
pH	7.38 (7.31–7.42)	7.39 (7.33–7.43)	7.36 (7.26–7.40)	0	0.077
Lactate, mg/dL	15.6 (10.9–27.0)	14.5 (10.4–24.7)	19.4 (13.0–31.8)	0	0.067
Hemoglobin, g/dL	12.3 (10.3–13.6)	12.6 (10.4–14.0)	11.8 (9.7–13.1)	0	0.081
Platelet, ×10^9^/L	180 (137–232)	181 (146–235)	155 (132–224)	1	0.333
Lactate dehydrogenase, U/L	473 (290–837)	424 (277–805)	679 (409–1080)	0	0.051
Aspartate aminotransferase, U/L	135 (38–372)	121 (36–371)	180 (71–497)	0	0.219
Alanine aminotransferase, U/L	40 (22–112)	39 (20–99)	63 (26–131)	0	0.167
Total bilirubin, mg/dL	0.7 (0.5–1.0)	0.7 (0.5–1.0)	0.9 (0.6–1.1)	1	0.261
Indirect bilirubin, mg/dL	0.6 (0.4–0.9)	0.6 (0.4–0.9)	0.7 (0.5–0.9)	2	0.469
Creatinine, mg/dL	0.97 (0.70–1.30)	0.92 (0.68–1.25)	1.08 (0.74–1.67)	1	0.101
Estimated glomerular filtration rate, mL/min	58.24 (39.53–79.14)	58.47 (43.84–82.62)	52.91 (31.48–76.04)	1	0.149
C‐reactive protein, mg/dL	0.34 (0.08–3.27)	0.34 (0.08–1.90)	0.39 (0.11–13.67)	1	0.187

Values are shown as n (%) or median (interquartile range). IABP indicates intra‐aortic balloon pumping; ICU, intensive care unit; MCS, mechanical circulatory support; and SCAI, Society for Cardiovascular Angiography and Interventions.

**Table 2:** Comparison of ICU Management and Clinical Outcomes of Patients Who Received IABP or Impella

	Overall	IABP	Impella	Missing	*P*‐value
Number	91	67	24		
Inotrope or vasopressor use					
Maximum vasoactive–inotropic score within 24 after ICU admission	0.00 (0.00–3.76)	0.00 (0.00–3.00)	3.00 (0.00–5.00)	1	0.072
No vasoactive support within the first 24 h	48 (53)	39 (59)	9 (38)	1	0.095
Time in therapeutic range for anticoagulation during MCS support					
ACT	50 (4–75)	50 (0–75)	50 (30–75)	11	0.418
Activated partial thromboplastin time	58 (20–89)	60 (25–83)	54 (5–97)	0	0.863
Catheter procedure and contrast use during ICU stay					
Coronary angiography	91 (100)	67 (100)	24 (100)	0	‐
Percutaneous coronary intervention	61 (67)	44 (66)	17 (71)	0	0.801
Culprit lesion–only percutaneous coronary intervention (n=61)	48 (79)	35 (80)	13 (76)	0	1.000
Enhanced computed tomography	14 (15)	11 (16)	3 (13)	0	0.753
Total volume of contrast media, mL	125 (85–180)	120 (70–180)	133 (104–195)	0	0.130
AKI during MCS support					
AKI (KDIGO stage 1–3)	47 (52)	30 (45)	17 (71)	0	0.034
Severity of AKI (n=47)				0	0.082
KDIGO stage 1	31 (66)	18 (60)	13 (76)		
KDIGO stage 2	10 (21)	7 (23)	3 (18)		
KDIGO stage 3	6 (13)	5 (17)	1 (6)		
Renal replacement therapy introduction	5 (5)	3 (4)	2 (8)	0	0.604
Time interval and outcomes					
MCS introduction to ICU admission interval, min	102 (82–140)	102 (71–135)	105 (92–178)	0	0.105
Duration of IABP or Impella support, day	2.83 (1.6–3.9)	2.7 (1.5–3.9)	3.0 (1.8–5.1)	0	0.331
Duration of venoarterial extracorporeal membrane support, d (n = 25)	2.4 (2.1–4.1)	2.3 (1.7–3.7)	3.0 (2.4–4.3)	0	0.164
Duration of mechanical ventilation, day (n = 63)	4.0 (1.9–7.0)	3.6 (1.9–6.6)	4.5 (2.2–7.7)	0	0.493
ICU stay, d	4.8 (2.6–8.2)	4.7 (2.4–6.1)	7.8 (4.3–9.4)	0	0.006
Hospital stay, d	26.1 (15.0–39.9)	25.3 (13.0–38.7)	33.8 (20.3–41.6)	0	0.068
ICU survival	80 (88)	58 (87)	22 (92)	0	0.721
Survival at discharge	69 (76)	50 (75)	19 (79)	0	0.785
30‐d survival after ICU admission	69 (76)	50 (75)	19 (79)	0	0.785

Values are shown as n (%) or median (interquartile range). ACT indicates activated clotting time; AKI, acute kidney injury; IABP, intra‐aortic balloon pump; ICU, intensive care unit; KDIGO, Kidney Disease: Improving Global Outcomes; and MCS, mechanical circulatory support.

Figure 1 shows the temporal changes in serum haptoglobin levels after ICU admission. Although haptoglobin levels at ICU admission did not differ significantly between the 2 groups, patients who received Impella exhibited a significantly greater reduction in haptoglobin beginning 12 hours after ICU admission compared with patients who received IABP (Figure 1A). This pattern remained consistent among patients who were not supported with concomitant V‐A ECMO support (Figure 1B). In contrast, among patients with concomitant V‐A ECMO support, haptoglobin levels remained persistently low in both groups, and no significant differences were observed (Figure 1C).

**Figure 1:**
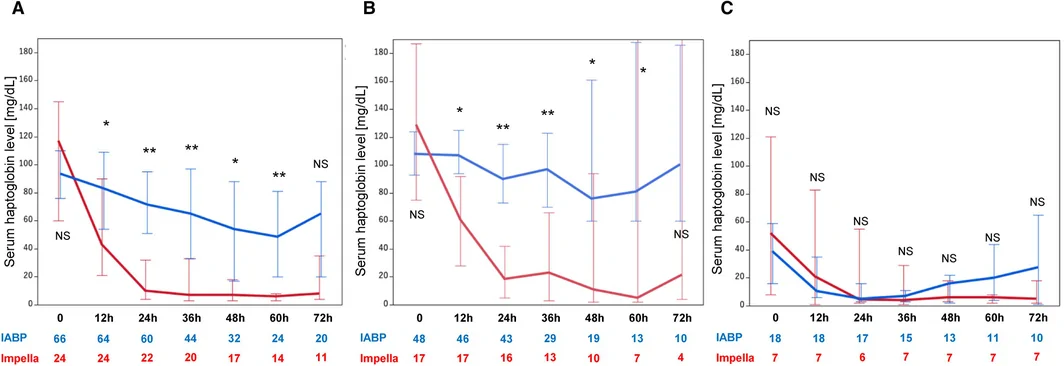
Temporal changes in serum haptoglobin levels after intensive care unit admission in patients who received IABP or Impella CP. **A**, All patients; (**B**) without concomitant venoarterial extracorporeal membrane oxygenation support; (**C**) with concomitant venoarterial extracorporeal membrane oxygenation support. Numbers below the *x* axis indicate the number of patients with available serum haptoglobin measurements at each time point. IABP indicates intra‐aortic balloon pump and NS, not significant, *P<0.05; **P<0.01.

The cumulative incidence of low haptoglobin levels and clinical hemolysis during device support is shown in Figure 2. Low haptoglobin occurred earlier and more frequently in patients supported with Impella than in those supported with IABP (log‐rank *P*=0.007). In contrast, the cumulative incidence of clinical hemolysis was low and did not differ significantly between the 2 groups (log‐rank *P*=0.685).

**Figure 2:**
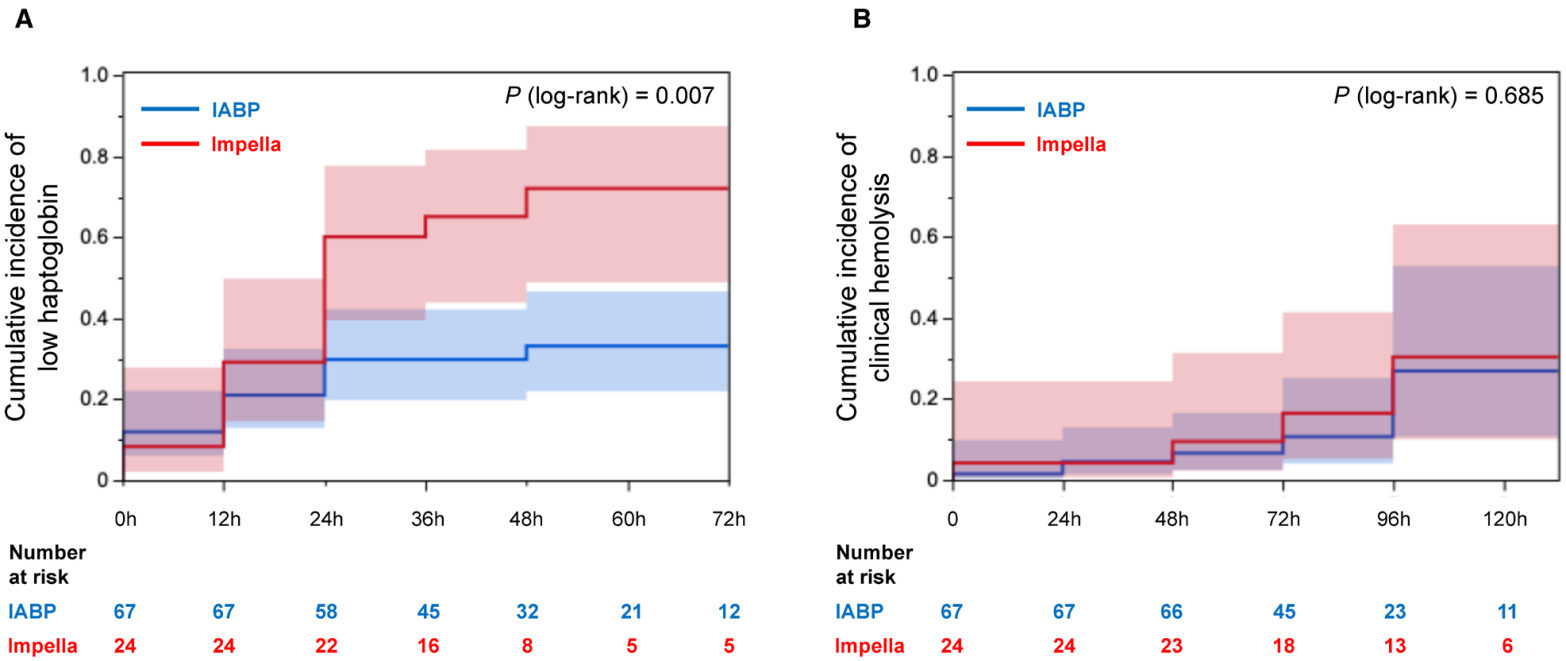
Cumulative incidence of low haptoglobin and clinical hemolysis after intensive care unit admission in patients who received IABP or Impella. **A**, Cumulative incidence of low haptoglobin levels (defined as <19 mg/dL). **B**, Cumulative incidence of clinical hemolysis (defined as low haptoglobin with total bilirubin >2.0 mg/dL). The shaded areas indicate the 95% CIs. *P* values were calculated using the log‐rank test. Patients without events were censored at the last available measurement during device support; measurements ended at device removal, death, or 7 days after intensive care unit admission, whichever occurred first. IABP indicates intra‐aortic balloon pump.

Figure S2 and Tables S1–S4 compare hemolysis‐related biomarkers. The median haptoglobin level during device support was significantly lower in the patients who received Impella than in those who received IABP. Low haptoglobin, defined as a serum haptoglobin level <19 mg/dL during device support, occurred significantly more frequently in patients who received Impella. Patients who received Impella also had significantly higher maximum levels of total bilirubin and lactate dehydrogenase.

Figure S3 and Tables S2 and S3 show the comparison stratified by concomitant V‐A ECMO use. Among patients without V‐A ECMO support (IABP, n=49; Impella, n=17), median haptoglobin levels during MCS were significantly lower in patients who received Impella compared with those who received IABP, and the proportion of patients with low haptoglobin was also significantly higher. The maximum total bilirubin and maximum lactate dehydrogenase levels were significantly higher in patients who received Impella. In contrast, among patients with concomitant V‐A ECMO support, both groups showed similarly depressed median haptoglobin levels during MCS, and no hemolysis‐related markers differed significantly between patients who received IABP and those who received Impella.

Firth's penalized logistic regression analysis showed that Impella use, concomitant V‐A ECMO support, and lower mean arterial pressure at ICU admission were significantly associated with low haptoglobin (Table 3).

**Table 3:** Firth's Penalized Logistic Regression Analysis of Predicting Low Haptoglobin During Device Support

	Odds ratio	95% CI	*P* value
Age, per y	0.99	0.95–1.09	1.000
Male sex	0.87	0.39–1.99	1.000
SCAI shock stage D or E before MCS introduction	0.78	0.34–1.63	0.487
Impella use (vs IABP use)	2.82	1.45–5.95	0.001
Concomitant V‐A ECMO support	16.52	4.65–174.56	<0.001
Mean arterial pressure at ICU admission, per mm Hg	0.95	0.89–0.99	0.006
Maximum alanine aminotransferase during MCS support, log‐transformed	1.44	0.83–2.74	0.200

IABP indicates intra‐aortic balloon pump; ICU, intensive care unit; MCS, mechanical circulatory support; SCAI, Society for Cardiovascular Angiography and Interventions; and V‐A ECMO, venoarterial extracorporeal membrane oxygenation.

In a sensitivity analysis restricted to patients with SCAI shock stages B through E, the main findings were generally consistent with the primary analysis, with Impella support showing a greater reduction in haptoglobin levels than IABP (Figure S4 and Table S4).

Tables S5 and S6 show the comparison of clinical outcomes and hemolysis‐related biomarkers according to low haptoglobin levels and clinical hemolysis during device support. Patients with low haptoglobin levels had a higher incidence of AKI and higher bilirubin levels. Although clinical hemolysis was not significantly associated with the overall incidence of AKI, it was associated with greater AKI severity, more frequent renal replacement therapy, and lower ICU survival.

## Discussion

In this single‐center retrospective study, we investigated hemolysis during IABP or Impella CP support using serial serum haptoglobin measurements and identified factors associated with its occurrence. Low haptoglobin was observed more frequently in patients receiving Impella than in those receiving IABP. Although baseline haptoglobin levels were similar, patients supported with Impella exhibited a marked decline beginning 12 hours after ICU admission, which persisted in the absence of concomitant V‐A ECMO. In contrast, when V‐A ECMO was used, haptoglobin levels were profoundly suppressed in both groups, with no significant differences between devices. Impella use, concomitant V‐A ECMO support, and lower mean arterial pressure at ICU admission were independently associated with low haptoglobin, whereas the incidence of overt clinical hemolysis did not differ between devices. The present study is novel in that it provides a direct comparison between Impella and IABP and further evaluates the impact of concomitant V‐A ECMO support. These findings demonstrate that the early decline in haptoglobin is more pronounced with Impella, particularly in the absence of V‐A ECMO, thereby providing a clinically relevant context for interpreting hemolysis during MCS. The similar incidence of clinical hemolysis despite the higher incidence of low haptoglobin with Impella may be explained by several factors. Low haptoglobin may capture mild or early hemolysis before progression to overt clinical hemolysis, although haptoglobin levels may also be influenced by nonhemolytic factors in critically ill patients. In addition, early recognition of declining haptoglobin may have prompted optimization of device management, potentially preventing progression to overt clinical hemolysis in some patients.

The DanGer Shock trial demonstrated improved survival with Impella CP in selected patients with infarct‐related CS^4^; however, subsequent analyses reported higher rates of AKI and renal replacement therapy in patients treated with Impella.^9^ AKI often developed early and was associated with suction events, higher pump performance levels, and longer support duration. Consistent with these findings, our previous single‐center study demonstrated a significant reduction in serum haptoglobin within 24 hours of Impella CP initiation, correlating with higher pump performance levels at ICU admission.^13^

These observations highlight hemolysis as a clinically relevant complication of Impella support and underscore the importance of early detection and careful device management to mitigate mechanical hemolysis and potentially reduce renal complications.^15^, ^16^, ^17^ Current international hemolysis criteria, such as those proposed by the MCS Academic Research Consortium, are based on assessments performed ≥72 hours after device initiation and may fail to capture early hemolysis.^18^ Moreover, lactate dehydrogenase, a commonly used marker of hemolysis, lacks specificity in the acute myocardial infarction setting due to concomitant myocardial injury.

In the present study, low serum haptoglobin occurred in 67% of Impella‐treated patients versus 31% of IABP‐treated patients. When combined with hyperbilirubinemia to define clinical hemolysis, the incidence was substantially lower and comparable between devices, particularly after excluding patients receiving concomitant V‐A ECMO. These findings align with prior reports demonstrating higher rates of subclinical hemolysis with Impella support,^5^, ^8^, ^19^, ^20^ while overt clinical hemolysis remains uncommon. Although AKI occurred more frequently with Impella, the incidence of severe AKI did not differ significantly between the groups. This may reflect limited statistical power, but it also suggests that serum haptoglobin is sensitive for detecting subclinical hemolysis that does not necessarily translate into severe renal dysfunction. Early monitoring using serial serum haptoglobin measurements may allow clinicians to avoid unnecessarily high pump settings and optimize adjunctive medical therapy. However, interventions based solely on serum haptoglobin levels may also have potential drawbacks. Reducing pump performance to mitigate hemolysis could compromise circulatory support and organ perfusion, and reliance on a single biomarker may lead to unnecessary treatment modifications. Therefore, serum haptoglobin should be interpreted in conjunction with the overall hemodynamic status and clinical context.

In patients supported with V‐A ECMO, hemolysis has been reported to occur in ≈5% to 18% of cases.^21^, ^22^ When hemolysis is defined as plasma‐free hemoglobin >50 mg/dL, the incidence has been reported to be ≈4%,^23^ whereas a lower threshold of >10 mg/dL yields substantially higher rates, up to 67%.^24^ Furthermore, patients receiving concomitant left ventricular unloading with devices such as IABP or Impella during V‐A ECMO support have been shown to experience significantly higher rates of hemolysis compared with those without unloading devices (46% versus 20%), while no significant difference in the need for renal replacement therapy was observed (31% versus 29%).^25^ Consistent with these observations, concomitant V‐A ECMO support was independently associated with low serum haptoglobin in the present study. However, the lack of a significant difference between the devices in the V‐A ECMO subgroup should be interpreted with caution. The relatively small sample size may have limited the statistical power to detect device‐related differences. Moreover, because V‐A ECMO itself is a strong contributor to hemolysis, it may have attenuated the differences between devices. These findings suggest that the apparent strong association between V‐A ECMO and hemolysis may, at least in part, reflect the severity of illness rather than a purely device‐specific effect. In this severely ill subgroup, the limited dynamic range of haptoglobin after depletion may also have reduced its ability to discriminate differences in hemolysis severity between devices.

While plasma‐free hemoglobin is considered a more direct marker of intravascular hemolysis and has been measured alongside haptoglobin in prior MCS studies,^15^, ^18^, ^20^ no established quantitative criteria exist to correlate specific haptoglobin or bilirubin levels with absolute plasma‐free hemoglobin concentrations in this population. Plasma‐free hemoglobin was not available at our institution. Haptoglobin is rapidly consumed through high‐affinity binding to free hemoglobin and may therefore serve as a practical marker of early hemolysis in routine clinical settings.^10^, ^26^, ^27^, ^28^ However, because its binding capacity is limited, once depleted it may no longer reflect differences in hemolysis severity, resulting in a floor effect.^28^ In addition, haptoglobin levels can be influenced by nonhemolytic factors such as hepatic dysfunction, systemic inflammation, infection, or nutritional status.^13^, ^27^, ^29^ Bilirubin, although commonly used as a supportive marker of hemolysis, may remain only mildly elevated in mechanical hemolysis and can also be affected by nonhemolytic factors such as shock‐related hepatic dysfunction or congestion.^12^, ^28^, ^30^ Therefore, the definitions used in the present study should be interpreted as pragmatic and clinically applicable rather than definitive. Further prospective studies incorporating plasma‐free hemoglobin are warranted to clarify the clinical significance of early hemolysis during MCS support.

This study has several limitations. First, as a retrospective, single‐center observational study, generalizability is limited, and causal inference cannot be made. Despite the use of Firth's penalized logistic regression to reduce small‐sample bias, the limited number of events relative to the number of covariates may still have resulted in model instability and potential overfitting. Therefore, the multivariable results should be interpreted as exploratory and supportive rather than confirmatory. Second, the number of patients treated with Impella CP was relatively small compared with those treated with IABP, and device selection was not randomized but determined by clinical judgment. Consequently, patients supported with Impella tended to present with more advanced CS, which may have influenced both hemolysis‐related biomarkers and clinical outcomes despite multivariable adjustment. Third, hemolysis was primarily assessed using serum haptoglobin. While haptoglobin is sensitive for detecting subclinical hemolysis, its levels can be affected by nonhemolytic factors such as hepatic dysfunction, systemic inflammation or infection, and nutritional status. Plasma‐free hemoglobin, which more directly reflects intravascular hemolysis, was not available at our institution and therefore could not be evaluated. As a result, the extent of mechanical hemolysis may have been under‐ or overestimated. In addition, carboxyhemoglobin, a by‐product of heme degradation, may serve as a quantitative indirect marker of hemolysis without the floor effect that limits haptoglobin. However, carboxyhemoglobin was not routinely measured at our institution and could not be evaluated in this study. Fourth, device‐specific factors potentially associated with hemolysis, including Impella pump performance level, suction alarms, catheter position, and detailed echocardiographic parameters, were not systematically analyzed. These factors may partly explain interindividual variability in hemolysis severity. Fifth, although our institution used a standardized protocol for suspected hemolysis, formal activation of the protocol was not consistently documented in this retrospective cohort. Therefore, we could not determine the exact number of patients in whom the protocol was activated or reliably distinguish hemolysis‐directed interventions from overall MCS management. In addition, the Kaplan–Meier analyses did not account for competing events, such as death or device removal, that could preclude subsequent development or detection of hemolysis‐related outcomes; therefore, these analyses should be interpreted as exploratory descriptions of temporal patterns during device support. Sixth, although Impella support was associated with a higher incidence of overall AKI, long‐term renal outcomes and functional recovery were not assessed. Therefore, the clinical implications of subclinical hemolysis detected by haptoglobin on long‐term prognosis remain uncertain. Finally, complete exchangeability between the treatment groups could not be ensured, and residual confounding due to confounding by indication remains a concern. To address this, we performed a sensitivity analysis restricted to patients with SCAI shock stages B through E, which yielded results generally consistent with the primary analysis.

In conclusion, in this single‐center retrospective study, hemolysis assessed by serial serum haptoglobin measurements occurred more frequently and earlier in patients supported with Impella CP than in those supported with IABP, particularly in the absence of concomitant V‐A ECMO. Although overt clinical hemolysis was uncommon and similar between devices, subclinical hemolysis was frequently observed during Impella support.

## Sources of Funding

None.

## Disclosures

S.K. reports honoraria/lecture fees from Abiomed, Getinge, Terumo, Tokai Medical Products, Mallinckrodt, Otsuka Pharmaceutical Co., Ltd., and Novartis Pharma K.K. The remaining authors have no disclosures to report.

## Supporting information

Figures S1–S4
Tables S1–S6

STROBE Statement Checklist S1
