# Supplementary material for: Early Decline in Serum Haptoglobin During Impella CP Support Compared With Intra‐Aortic Balloon Pump in Cardiogenic Shock: A Single‐Center Observational Study
Source: J Am Heart Assoc. 2026 Aug 7;15(16):e049038. doi: 10.1161/JAHA.126.049038 (PMC13695740; doi:10.1161/JAHA.126.049038)

SUPPLEMENTAL MATERIAL

**Table S1: The comparison of hemolysis-related biomarkers between patients who received intra-aortic balloon pumping and Impella during device support**	**Overall**	**IABP**	**Impella**	**Missing**	**P-value**
**Number**	91	67	24		
Median haptoglobin, mg/dL	64 (13-112)	74 (22-124)	22 (4-61)	0	0.002
Minimum haptoglobin <19mg/dL	37 (41)	21 (31)	16 (67)	0	0.004
Maximum aspartate aminotransferase, U/L	220 (64-535)	167 (61-535)	253 (73-541)	0	0.290
Maximum alanine aminotransferase, U/L	56 (26-138)	44 (25-127)	73 (30-155)	0	0.412
Maximum lactate dehydrogenase, U/L	736 (426-1375)	692 (407-1172)	1011 (645-1786)	0	0.045
Maximum lactate dehydrogenase >1000U/L	33 (36)	21 (31)	12 (50)	0	0.103
Maximum total bilirubin, mg/dL	1.1 (0.8-1.6)	1.0 (0.8-1.6)	1.3 (1.0-2.0)	0	0.030
Maximum total bilirubin >=2.0mg/dL	17 (19)	11 (16)	6 (25)	0	0.371
Maximum indirect bilirubin, mg/dL	0.9 (0.7-1.2)	0.8 (0.6-1.1)	1.0 (0.8-1.4)	0	0.035
Clinical hemolysis	11 (12)	7 (10)	4 (17)	0	0.472

IABP, intra-aortic balloon pumping

**Table S2: Comparison of hemolysis-related biomarkers between patients who received intra-aortic balloon pumping and Impella without concomitant veno-arterial extracorporeal membrane oxygenation**	**IABP only**	**Impella only**	**P-value**
**Number**	**49**	**17**	
Median haptoglobin, mg/dL	103 (69-138)	41 (7-81)	<0.001
Minimum haptoglobin <19mg/dL	4 (8)	9 (53)	<0.001
Maximum aspartate aminotransferase, U/L	88 (50-243)	248 (56-418)	0.173
Maximum alanine aminotransferase, U/L	37 (22-104)	56 (28-128)	0.308
Maximum lactate dehydrogenase, U/L	487 (359-774)	808 (524-1538)	0.019
Maximum lactate dehydrogenase >1000U/L	8 (16)	7 (41)	0.048

Maximum total bilirubin, mg/dL	0.9 (0.7-1.2)	1.3 (1.0-1.9)	0.024
Maximum total bilirubin >=2.0mg/dL	5 (10)	4 (24)	0.220
Maximum indirect bilirubin, mg/dL	0.8 (0.5-1.0)	0.9 (0.7-1.3)	0.114
Clinical hemolysis	1 (2)	2 (12)	0.161

IABP, intra-aortic balloon pumping

**Table S3: Comparison of hemolysis-related biomarkers between patients who received intra-aortic balloon pumping and Impella with concomitant veno-arterial extracorporeal membrane oxygenation**	**IABP with ECMO**	**Impella with ECMO**	**P-value**
**Number**	**18**	**7**	
Median haptoglobin, mg/dL	11 (4-24)	10 (3-24)	0.545
Minimum haptoglobin <19mg/dL	17 (94)	7 (100)	1.000
Maximum aspartate aminotransferase, U/L	610 (372-687)	535 (150-1274)	0.904
Maximum alanine aminotransferase, U/L	116 (80-174)	112 (34-274)	0.809
Maximum lactate dehydrogenase, U/L	1517 (904-1948)	2320 (636-4810)	0.468
Maximum lactate dehydrogenase >1000U/L	13 (72)	5 (71)	1
Maximum total bilirubin, mg/dL	1.4 (0.8-2.7)	1.3 (1.1-2.2)	0.927
Maximum total bilirubin >=2.0mg/dL	6 (33)	2 (29)	1.000
Maximum indirect bilirubin, mg/dL	1.1 (0.6-2.3)	1.1 (1.0-1.3)	0.739
Clinical hemolysis	6 (33)	2 (29)	1.000

IABP, intra-aortic balloon pumping; ECMO, extracorporeal membrane oxygenation

**Table S4: Comparison of clinical characteristics, outcomes, and hemolysis-related biomarkers between intra-aortic balloon pumping and Impella in patients with SCAI stage B–E (sensitivity analysis)**	**IABP**	**Impella**	**P-value**
**Number**	**45**	**23**	
**Patient characteristics**			
Age	74 (64-79)	75 (71-78)	0.795

Male	35 (78)	18 (78)	1.000
Acute myocardial infarction	31 (69)	22 (96)	0.013
**Patient status before MCS introduction**			
SCAI shock stage			0.404
B	11 (24)	4 (17)	
C	11 (24)	4 (17)	
D	9 (20)	9 (39)	
E	14 (31)	6 (26)	
pH	7.33 (7.19-7.41)	7.33 (7.27-7.41)	0.796
Lactate, mg/dL	35.5 (21.1-66.1)	27.4 (19.2-69.5)	0.873
Glasgow Coma Scale <8	21 (47)	3 (13)	0.007
Concomitant veno-arterial extracorporeal membrane support	18 (40)	7 (30)	0.596
**Patient status at ICU admission**			
Mechanical ventilation	33 (73)	20 (87)	0.235
Mean arterial pressure, mmHg	80 (68-95)	78 (71-87)	0.595
Heart rate, beats per minute	83 (71-101)	100 (85-108)	0.046
Maximum vasoactive-inotropic score within 24 hours after ICU admission	2.09 (0.00-4.75)	3.00 (0.00-5.00)	0.637
**Clinical outcomes**			
Acute kidney injury (KDIGO stage 1-3)	26 (58)	16 (70)	0.433
Severity of acute kidney injury (n=41)			0.339
KDIGO stage 1	14 (54)	12 (75)	
KDIGO stage 2	7 (27)	3 (19)	
KDIGO stage 3	5 (19)	1 (6)	
Renal replacement therapy introduction	3 (7)	2 (9)	1.000
ICU survival	36 (80)	21 (91)	0.310
30-day survival after ICU admission	29 (64)	18 (78)	0.281
**Hemolysis-related biomarkers**			
Median haptoglobin, mg/dL	55 (11-112)	19 (4-62)	0.057
Minimum haptoglobin <19mg/dL	19 (42)	16 (70)	0.042
Maximum total bilirubin, mg/dL	1.1 (0.8-1.9)	1.3 (1.0-2.1)	0.194
Clinical hemolysis	7 (16)	4 (17)	1.000

SCAI, Society for Cardiovascular Angiography and Interventions; IABP, intra-aortic

balloon pumping; MCS, mechanical circulatory support; ICU, intensive care unit; KDIGO, Kidney Disease: Improving Global Outcomes.

**Table S5: Comparison of clinical outcomes and hemolysis-related biomarkers between patients with low and normal haptoglobin during mechanical circulatory support**	**Haptoglobin <19mg/dL**	**Haptoglobin ≥19mg/dL**	**P-value**
**Number**	**37**	**54**	
Acute kidney injury (KDIGO stage 1-3)	26 (70)	21 (39)	0.003
Severity of acute kidney injury (n=47)			0.010
KDIGO stage 1	15 (58)	16 (76)	
KDIGO stage 2	6 (23)	4 (19)	
KDIGO stage 3	5 (19)	1 (5)	
Renal replacement therapy introduction	4 (11)	1 (2)	0.236
Maximum total bilirubin, mg/dL	1.4 (1.1-2.3)	0.9 (0.7-1.2)	<0.001
Maximum total bilirubin >=2.0mg/dL	11 (30)	6 (11)	0.031
Maximum indirect bilirubin, mg/dL	1.1 (0.8-1.4)	0.8 (0.5-1.0)	<0.001
Clinical hemolysis	11 (30)	0 (0)	<0.001
ICU survival	27 (73)	53 (98)	<0.001
30-day survival after ICU admission	20 (54)	49 (91)	<0.001

KDIGO, Kidney Disease: Improving Global Outcomes; ICU, intensive care unit.

**Table S6: Comparison of clinical outcomes and hemolysis-related biomarkers between patients with and without clinical hemolysis during mechanical circulatory support**	**With clinical hemolysis**	**Without clinical hemolysis**	**P-value**
**Number**	**11**	**80**	
Acute kidney injury (KDIGO stage 1-3)	9 (82)	38 (48)	0.051
Severity of acute kidney injury (n=47)			<0.001
KDIGO stage 1	3 (33)	28 (74)	
KDIGO stage 2	2 (22)	8 (21)	
KDIGO stage 3	4 (44)	2 (5)	
Renal replacement therapy introduction	3 (27)	2 (3)	0.012

ICU survival	6 (55)	74 (93)	0.003
30-day survival after ICU admission	6 (55)	63 (79)	0.127

KDIGO, Kidney Disease: Improving Global Outcomes; ICU, intensive care unit.

Figure S1: Conceptual diagram between low haptoglobin and key predictors in this study.

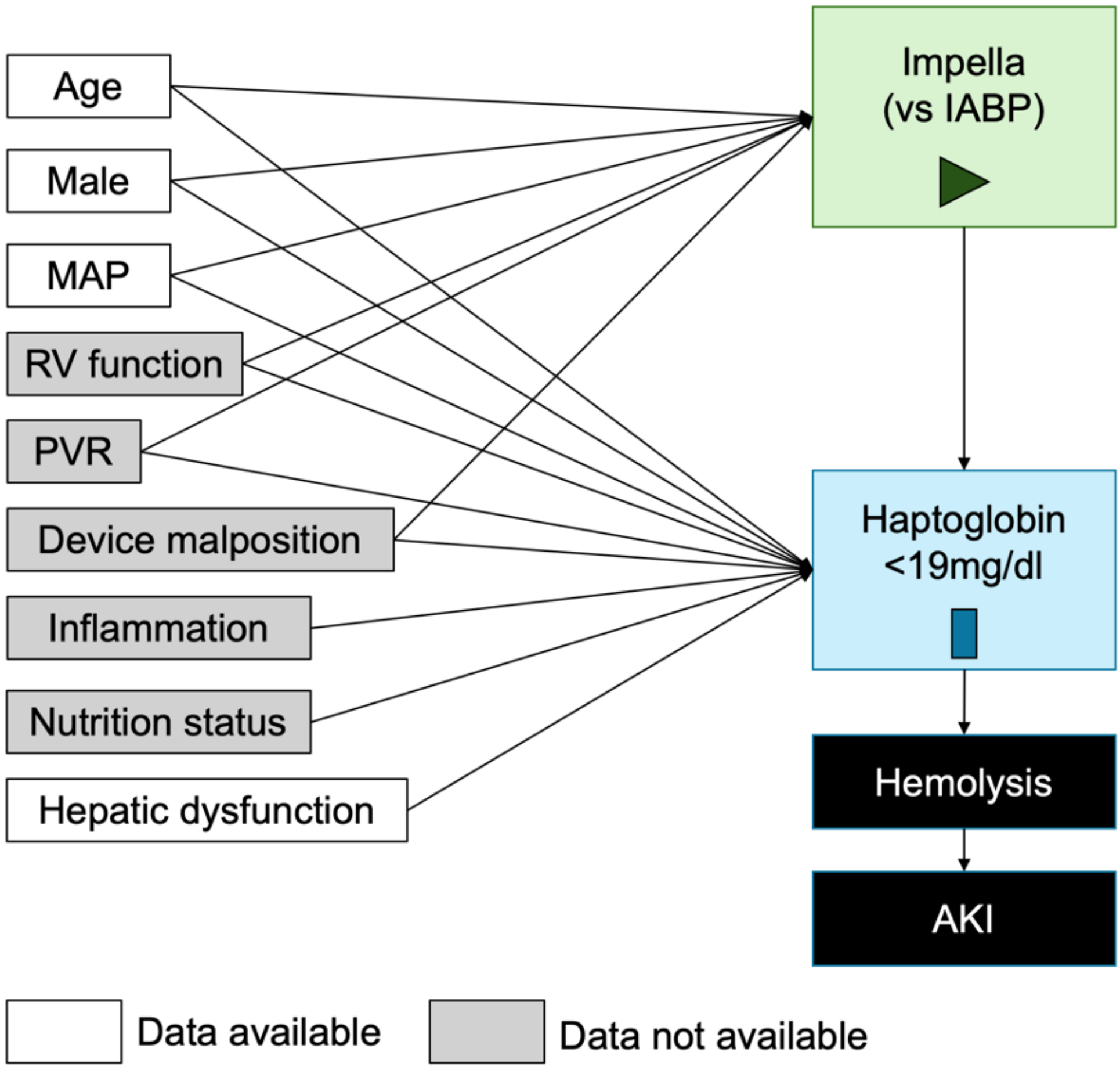

MAP, mean arterial pressure; RV, right ventricular; PVR, pulmonary vascular resistance; AKI, acute kidney injury.

Figure S2: Comparison of hemolysis-related biomarkers between patients who received IABP and Impella during device support, including (A) median haptoglobin levels, (B) proportion of low haptoglobin, (C) maximum total bilirubin levels, and (D) maximum lactate dehydrogenase levels.

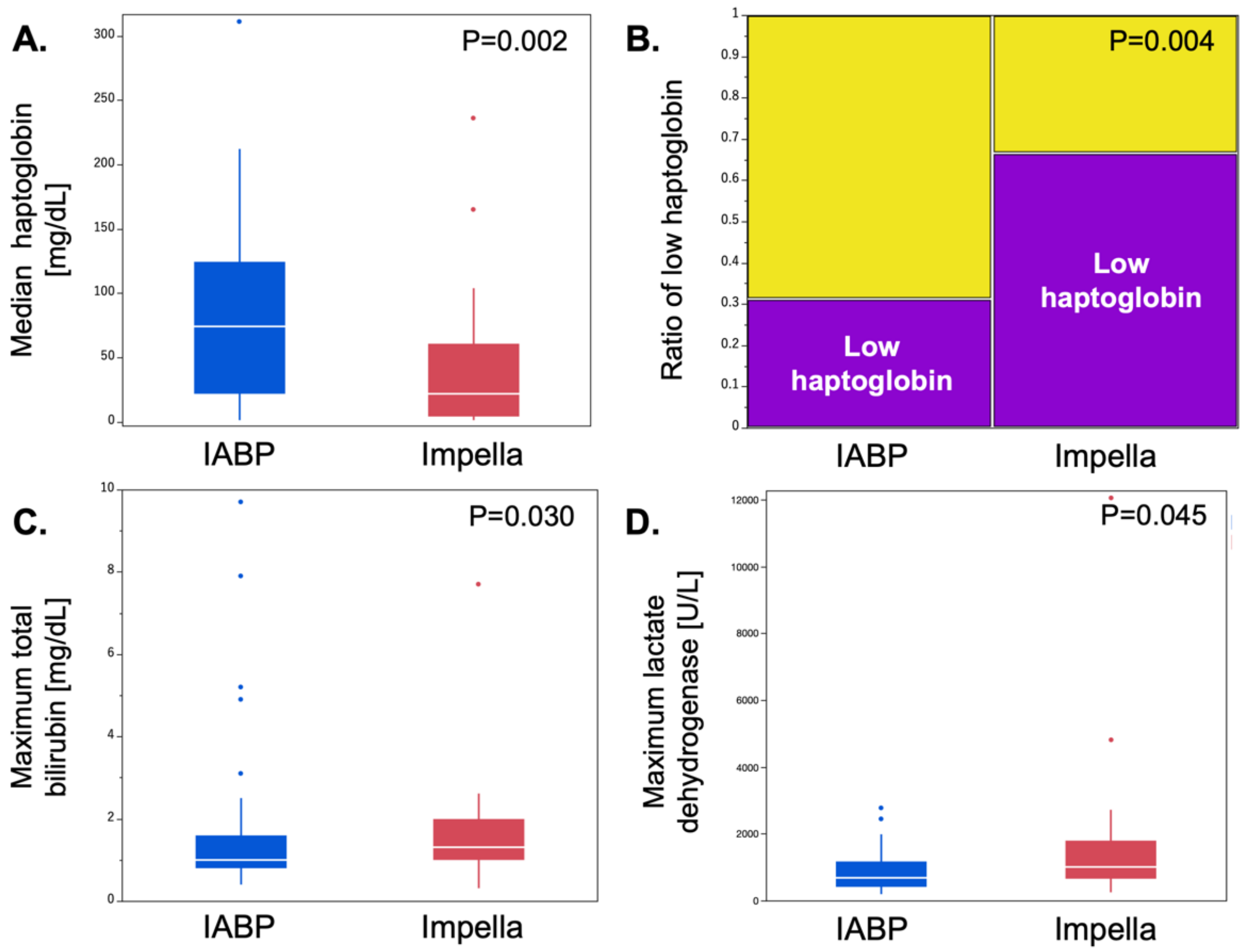

Figure S3: Comparison of hemolysis-related biomarkers between patients who received intra-aortic balloon pumping and those who received Impella, stratified by concomitant veno-arterial extracorporeal membrane oxygenation support. Panels present (A) median haptoglobin levels, (B) proportion of low haptoglobin, (C) maximum total bilirubin, and (D) maximum lactate dehydrogenase levels.

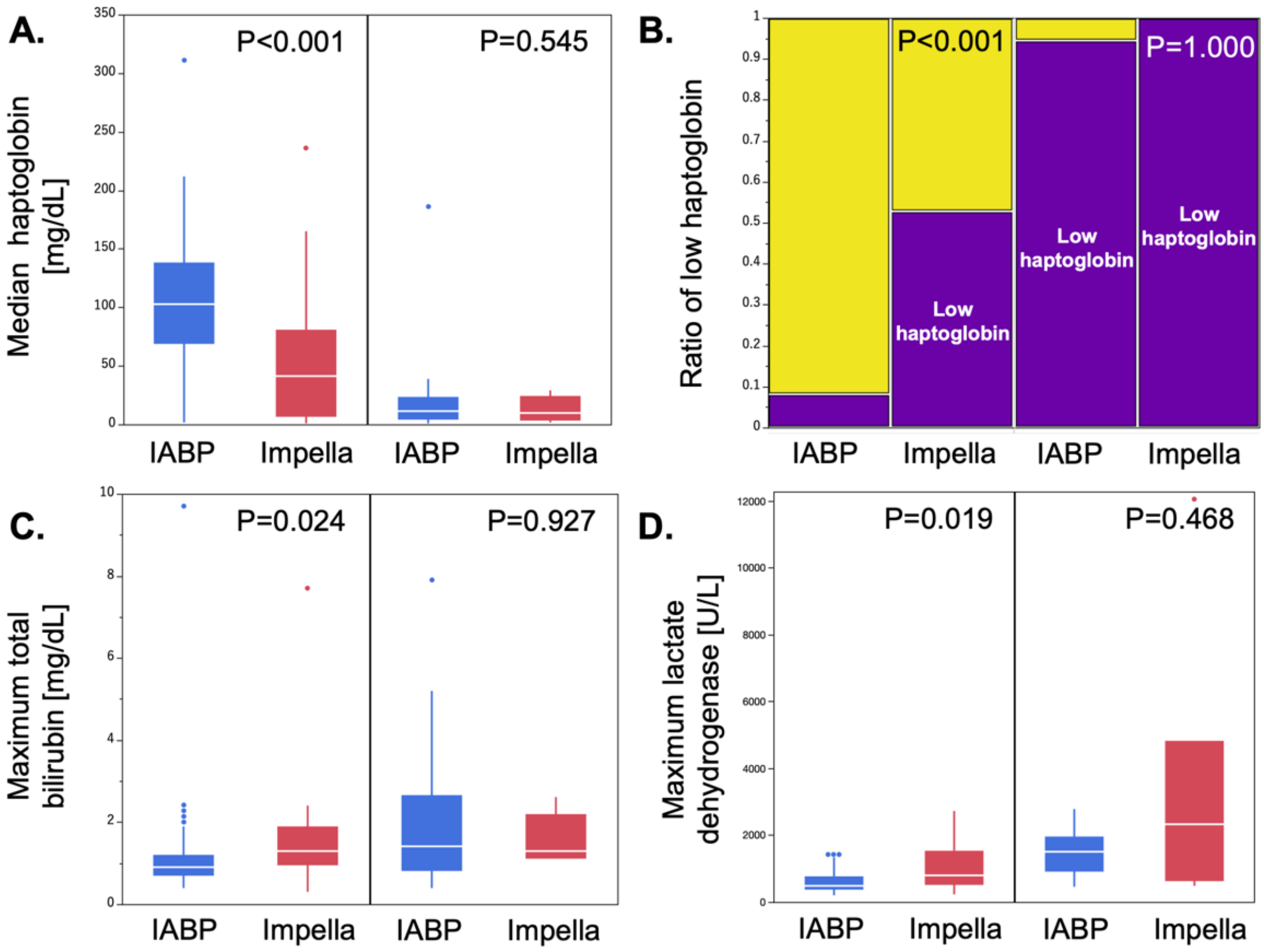

Figure S4: Temporal changes in serum haptoglobin levels after intensive care unit admission in patients with SCAI B–E who received intra-aortic balloon pumping or Impella (sensitivity analysis). (A) all patients; (B) without concomitant veno-arterial extracorporeal membrane oxygenation support; (C) with concomitant veno-arterial extracorporeal membrane oxygenation support. *P < 0.05; **P < 0.01; NS, not significant.

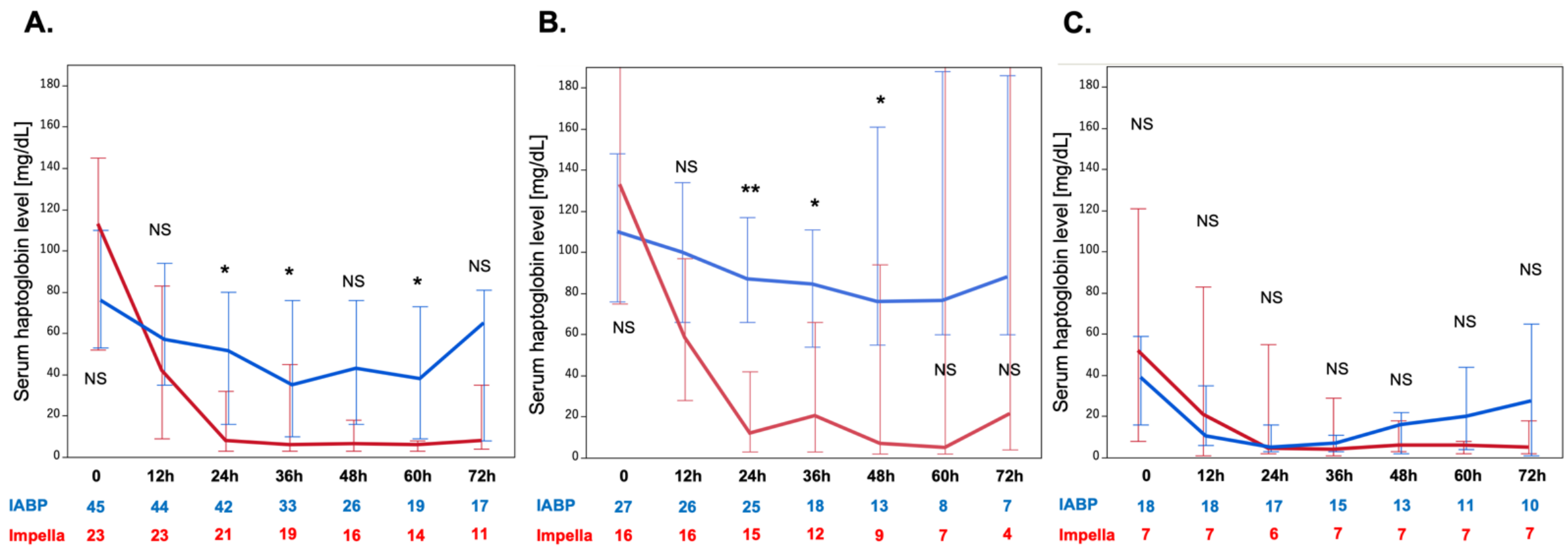

Supplement: Supplementary file 1 [file JAH3-15-e049038-s001.pdf]
